# A Paradigm for Peptide Hormone-GPCR Analyses

**DOI:** 10.3390/molecules25184272

**Published:** 2020-09-18

**Authors:** Fred Naider, Jeffrey M. Becker

**Affiliations:** 1 Department of Chemistry, College of Staten Island, CUNY, 2800 Victory Blvd, Staten Island, NY 10314, USA; 2 Department of Microbiology, University of Tennessee, 610 Ken and Blaire Mossman Building, 1311 Cumberland Avenue, Knoxville, TN 37996, USA

**Keywords:** peptide pheromone, G protein-coupled receptors, nuclear magnetic resonance, photoactivated crosslinking, chemical crosslinking, receptor mutation, peptide analogs, *Saccharomyces cerevisiae*, fluorescence screening, receptor-ligand interaction

## Abstract

Work from our laboratories over the last 35 years that has focused on Ste2p, a G protein-coupled receptor (GPCR), and its tridecapeptide ligand α-factor is reviewed. Our work utilized the yeast *Saccharomyces cerevisiae* as a model system for understanding peptide-GPCR interactions. It explored the structure and function of synthetic α-factor analogs and biosynthetic receptor domains, as well as designed mutations of Ste2p. The results and conclusions are described using the nuclear magnetic resonance interrogation of synthetic Ste2p transmembrane domains (TMs), the fluorescence interrogation of agonist and antagonist binding, the biochemical crosslinking of peptide analogs to Ste2p, and the phenotypes of receptor mutants. We identified the ligand-binding domain in Ste2p, the functional assemblies of TMs, unexpected and interesting ligand analogs; gained insights into the bound α-factor structure; and unraveled the function and structures of various Ste2p domains, including the N-terminus, TMs, loops connecting the TMs, and the C-terminus. Our studies showed interactions between specific residues of Ste2p in an active state, but not resting state, and the effect of ligand activation on the dimerization of Ste2p. We show that, using a battery of different biochemical and genetic approaches, deep insight can be gained into the structure and conformational dynamics of GPCR-peptide interactions in the absence of a crystal structure.

## 1. Introduction

The superfamily of heptahelical membrane receptors has been a major focus of research activity for more than 30 years. These receptors, often known as G protein-coupled receptors (GPCRs) when coupled with a G protein, are ubiquitous in eukaryotic cells occurring throughout the animalia, plantae, and fungal kingdoms and are involved in many human pathologies. Accordingly, either serendipitously or by design they are the targets of many pharmaceuticals. Although many pharmaceuticals were developed without the knowledge of their molecular targets, a full rational design actually requires an atomic-level understanding of the binding sites and mechanism of action. Therefore, a great deal of experimentation was focused on finding the structures of these receptors. Based on the availability of highly purified rhodopsin from eye tissue, a high-resolution structure of this membrane receptor was solved in 2000. It took about a decade more to solve the structure of the β-adrenergic receptor using modern cloning, expression, and X-ray technologies. Today, hundreds of structures of GPCRs bound to antagonists, agonists, allosteric modulators, and other elements of the signal transduction machinery may be found in the protein data bank, and investigators using cryo-electron microscopy are elucidating even more complex assemblies. Based on DNA sequencing, it is estimated that there are about 800 GPCRs in the human genome alone.

The huge science of GPCRs remains a fertile playground for understanding the subtle factors that are at the heart of biological specificity. For example, we all know that dogs have an acute sense of smell that can distinguish differences unfathomable to humans. Is this related to the number of olfactory GPCRs (OR), their affinities, the density of their expression in the nasal epithelium, or the receptor specificity? It is unlikely, given the extremely large number of OR receptors both in humans and in dogs, that this issue will be solved at the structural level. Other complementary approaches are needed.

Our laboratories have studied the interaction of peptides with cells and cell membranes since the early 1970s. Initially, we explored the way that peptides are actively concentrated by eukaryotic cells. Using the yeast *Saccharomyces cerevisiae* as the test organism, we studied the structural specificity and regulation of peptide transporters, culminating in the discovery of two classes of peptide transporters in fungi and plants: the PTR (a system for the transport of di- and tri-peptides) and OPT (a system for longer length peptides) families [1]. Given our interest in peptide-yeast interactions, our focus evolved to understanding how haploid yeast cells communicate, leading to a broad exploration of the mechanism of action of the yeast α-mating factor and, to a lesser extent, of the yeast **a**-factor. This research, which commenced in the early 1980s, resulted in a paradigm for investigations into yeast mating and, in particular, to studies on the biology, chemistry, biochemistry, and biophysics of how a medium-sized peptide interacted with its cognate GPCR. Along the way, we developed and applied methods to assay this system, to synthesize peptide analogs that were powerful probes for the functional domains of the ligand and to crosslink the ligand into the receptor, thereby identifying the receptor binding site. In parallel, we initiated genetic, biochemical, and biophysical studies on the structure of the Ste2p, the GPCR that recognized the α-factor. Although one might argue that these studies have now been supplanted by the ability of crystallographers, electron microscopists, and nuclear magnetic resonance (NMR) spectroscopists to study an intact GPCR and its interaction with ligands, our studies provided a roadmap showing the role of peptide surrogates in understanding the structure and biophysical characteristics of a particular GPCR. This chapter provides an overview of our studies. We acknowledge the studies of many other investigators who added greatly to an understanding of the α-factor-Ste2p system. We refer the reader to a recent comprehensive review of *S. cerevisiae* and its pheromone-receptor-signal transduction system as a paradigm for understanding GPCR-initiated signaling and its regulation [2]. When relevant to our modest contributions, the findings of those investigations are cited in this review.

## 2. The Mating Pathway in the Yeast *Saccharomyces cerevisiae*: Relevance to GPCRs

Sexuality in the budding yeast *Saccharomyces cerevisiae* involves the joining, called conjugation in this context, of haploid cells of opposite mating types, designated alpha (*MATα*) and **a** (*MAT**a***), respectively. A diffusible factor, called α-factor, secreted by *MATα* cells and required for mating was discovered in 1973 from a culture broth of *MATα* cells [3]. This factor and its identical chemically synthesized equivalent [4,5] is a 13-amino acid peptide. Although studies from our lab and those of others started to determine the structure-function relationships of the pheromone [6,7], how this secreted peptide controlled mating in yeast was not revealed until the genes involved in the mating process were identified about 10 years later.

**Figure 1:**
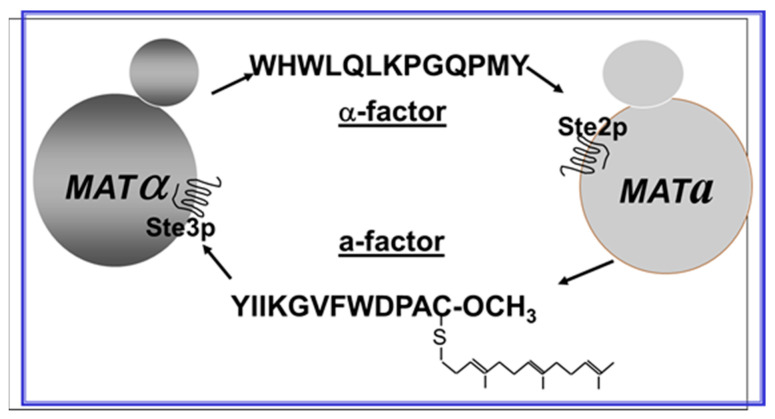
A diagrammatic representation of the pheromones (α-factor and **a**-factor) mediating the sexual conjugation of *Saccharomyces cerevisiae* haploid cells of opposite mating types (*MATα* and *MAT**a***). *MATα* and *MAT**a*** cells secrete their cognate pheromones to prepare their opposite mating type for mating (conjugation), resulting in a diploid cell. Ste2p and Ste3p are the membrane-bound receptors on *MAT**a*** and *MATα* cells, respectively, that recognize their bound pheromones to arrest the recipient cell in the G1 phase of the cell cycle in preparation for conjugation. Pheromones and hormones perform physiologically equivalent functions; pheromones act between individuals of the same species, whereas hormones are produced and act within an individual.

A catalogue of mutations in specific genes generated in the Hartwell lab, called *STERILE* or *STE*, yielded yeast strains that could not mate [8]. These mutants were the key to identifying cellular proteins encoded by the *STE* genes involved in conjugation, thus revealing the receptors and cellular response pathways necessary for haploid yeast cells to mate (see Figure 1 for a diagram of the role of peptide pheromones and their receptors in preparation for sexual conjugation in *S. cerevisiae*).

**Figure 2:**
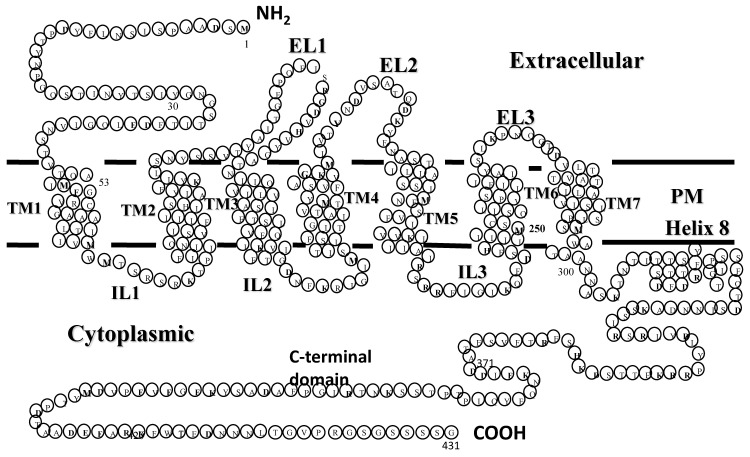
Snake diagram of Ste2p. A 2D representation of Ste2p showing the N-terminus, seven-transmembrane TM bundle, C-terminus (CT), and three extracellular (EL) and three intracellular (IL) loops that connect TMs to each other. The TMs are embedded in the plasma membrane (PM), with the N-terminus facing extracellularly and the C-terminus facing intracellularly. The presence of an 8th helix is indicated, as will be discussed later in the review.

Experiments using radiolabeled versions of the α-factor indicated that a cellular receptor encoded by the *STE2* gene was involved in transmitting a signal started by pheromone binding to initiate a cellular response necessary for the mating interaction [9,10,11]. The cloning of *STE2* and analysis of its encoded protein showed it was a seven-transmembrane protein of 431 amino acids (see Figure 2 for “Snake Diagram of Ste2p” [12,13]). At the time of its cloning, there was no other GPCR in the protein databank. Thus, Ste2p (the receptor of α-factor encoded by the *STE2* gene) was the first GPCR cloned. About a year later, the β-adrenergic receptor, a mammalian GPCR, was cloned [14], starting an explosion of the study of GPCRs—a GPCR revolution.

## 3. Peptide Ligands for Ste2p, the Yeast GPCR for the Peptide Pheromone α-Factor

### 3.1. The Tridecapeptide α-Factor Has Functional Domains

The availability of *S. cerevisiae* to investigate cell-cell communication and intracellular signaling, together with the advanced state of yeast genetics, provided a powerful paradigm with which to study the molecular and biochemical processes involved in GPCR activity. Many laboratories chose to use this model system. One of our specific goals was to use peptide synthesis together with yeast bioassays and biochemistry to understand the function and activation of Ste2p as a model GPCR [2,15,16,17]. Much of our work, and those of others, on the structure-activity relationship of α-factor has been reviewed previously [7]. In the next section, we will summarize how these studies led to insights into the interaction of the α-factor with Ste2p and to the discovery that the pheromone had distinct domains that made major contributions to the binding and activation of the receptor.

The tridecapeptide α-factor (WHWLQLKPGQPMY) was shown to be biosynthesized as a precursor “pre-prohormone” that was enzymatically processed to the final, biologically active peptide pheromone [18]. Our early studies on α-factor involved the development of synthetic approaches to efficiently prepare analogs and derivatives of the pheromone. Initially, we used solution phase synthesis to prepare dodecapeptide analogs because this was one of the active forms originally isolated from growth cultures [4]. The synthesis of a great variety of analogs was eventually carried out by solid-state approaches, as it was more efficient than solution phase strategies.

An alanine-scanning investigation clarified the fact that the activation of Ste2p was primarily due to residues near the N-terminus of the pheromone, whereas residues at the carboxyl terminus played a role primarily in binding [19]. Studies using double mutant cycle scanning supported the importance of the α-factor N-terminus in interactions with Ste2p residues N205 and Y266 that were critical for receptor signaling [20]. The involvement of residues N205 and Y266 in receptor function will be discussed in more detail in subsequent sections of this review. Together, these studies suggested that the 13-residue peptide pheromone might activate in a two-step process. First, the carboxyl end of α-factor binds to the receptor, and then, provided that the peptide can assume the correct conformation (see below), the N-terminus binds and triggers activity. This hypothesis, first put forth in the early 2000s, seems to be highly relevant to GPCR activation by various peptides. For example, a two-step (likely even more complicated than two steps) mechanism of signal activation is now widely accepted for the chemokine activation of chemokine receptors [21] and the glucagon-like peptide receptor [22,23], and it may reflect an evolutionary conserved mechanism, in that a simple unicellular yeast seems to have an activation mechanism related to that of mammalian cells. In fact, the human hypothalamic decapeptide gonadotropin-releasing hormone (GnRH) and α-factor are evolutionarily similar, and the α-factor activated cultured gonadotrophs. [24].

### 3.2. Conformation of α-Factor at the Step Binding Site

The linear tridecapeptide α-factor was not expected to form a structured molecule in aqueous solution. However, when bound to Ste2p it must assume the “biologically active structure”. Circular Dichroism (CD) studies by us and others indicated that, in water or buffer, both the 12- and 13-residue α-factor peptide assumed a distribution of structures [25,26]. However, similar studies in organic aqueous mixtures and in the presence of detergent or membrane lipids gave CD spectra that indicated a change in the conformational distribution assumed by the pheromone. Most strikingly, there was a weak correlation between the CD spectra and the residue in position 9 of α-factor. The native pheromone has a Pro-Gly sequence which is highly associated with a turn, specifically a Type II β-turn. Such a structure is known to be stabilized by the insertion of a d-residue in place of Gly and destabilized by the replacement of Gly with an l-residue. Indeed, the d-Ala^9^-α-factor had the same biological activity and affinity as α-factor, whereas the l-Ala^9^-α-factor was almost inactive [19]. Moreover, NMR spectroscopy supported the assumption of a Type-II β-turn in the d-Ala^9^ but not in the l-Ala^9^-α-factor [27,28,29]. It was exciting to discover that the formation of this turn was favored in the presence of lipid [28,29]. Parallel studies from the Sakakibara group concluded that, when bound to membranes, the tridecapeptide α-factor assumed a 3_10_-helix at the N-terminus, followed by turn structures in the middle and end of the pheromone [30,31,32]. These findings were consistent with the hypothesis of Schwyzer [33] that membrane lipids capture many peptide hormones and provide an environment that both aids their diffusion to the receptor and stabilizes their native conformation. We note that the crystal structures reveal that the binding sites of many GPCRs do seem to be mostly in the portion of the seven-helix bundle that is close to the extracellular face of the membrane. This would suggest that ligands might just transfer from the aqueous environment to the binding pocket. However, Bader and Zerbe have shown that the pancreatic polypeptide binds to an extracellular region of the NY4 receptor and between a putative amphiphilic helix in the N-terminus and the first transmembrane domain [34]. They hypothesize that this would be consistent with Schwyzer’s work on the role of the membrane in peptide hormone activity and propose that the receptor N-terminus guides the peptide to the ligand binding site by a “fly-casting” mechanism [34].

**Figure 3:**
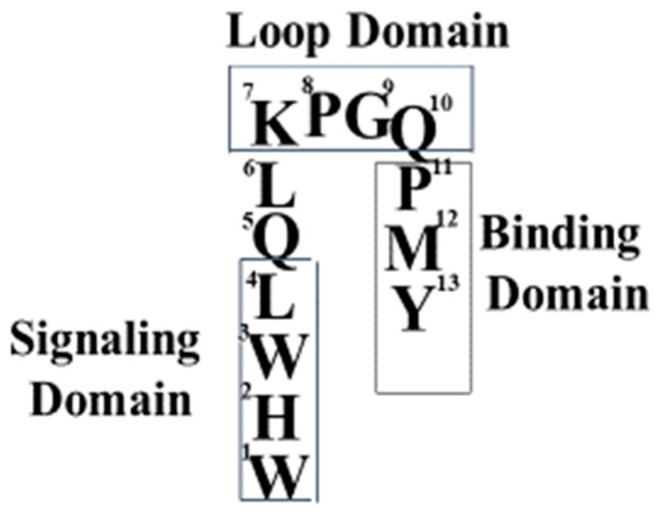
Biologically active conformation of α-factor. The tridecapeptide is flexible around residues 7–10, which we believe form a β-turn-like loop. After the three C-terminal residues of the peptide bind to Ste2p, the loop region allows the “bending” of α-factor to form a conformation in which the three N-terminal residues productively interact with Ste2p to initiate signal transduction.

The Sakakibara group also gave evidence that the N- and C-termini of the pheromone were near to each other. Synthetic α-factor analogs with a covalent cyclic ring in the center of the peptide were found to be weak agonists [35,36]. Together, the biophysical studies on various α-factor analogs led us to conclude that the biologically active structure of α-factor bound to its GPCR involved a turn in the middle of the pheromone, whose function was to allow the proper orientation of the N and C- terminal ends of the pheromone in the active site of Ste2p (see Figure 3 for a diagram of the pheromone active conformation).

### 3.3. Discovery of Synergists, Allosteric Ligands, and Biased Agonists

During our studies of α-factor and Ste2p over the course of 35 years, we have made some observations that could not have been predicted. Of course, such serendipity is common to most scientific investigations. Yet, these discoveries added greatly to our interest and excitement in probing the mysteries of GPCRs using mating in yeast as our paradigm. We describe below three of these findings that resulted from our work with α-factor analogs.

#### 3.3.1. Synergists/Allosteric Ligands

We found that biologically inactive, truncated analogues of α-factor either antagonized or increased the activity of the native pheromone [37]. Amino-terminal truncated pheromones desTrp^1^, desHis^2^-α-factor and desTrp^1^[Ala^3^]α-factor had no activity by themselves, but these analogues acted as antagonists by competing with the binding and activity of the mating factor. In contrast, a carboxyl-terminal truncated pheromone desTyr^13^, desMet^12^-α-factor was not active by itself, nor did the peptide compete with α-factor for binding to Ste2p. However, this latter peptide caused a marked increase in the α-factor potency. We termed this phenomenon synergy, but now realize this could be an example of an allosteric interaction.

It has been recognized that allosteric modulators can regulate GPCR activity by binding to the receptor at sites distinct from, or overlapping with, those occupied by the orthosteric ligand [38]. An inadvertent finding showed that yeast was sensitive to novobiocin, a coumarin drug, and that the effect of the drug was mediated through Ste2p [39]. This established novobiocin as a agonist of Ste2p. Studies in the Konopka lab [40] corroborated the finding that novobiocin is a weak agonist by showing the growth arrest of yeast by novobiocin. We demonstrated that both novobiocin and an 11 amino acid, c-terminally truncated α-factor analog ([desMet^12^, desTyr^13^]α-factor), were positive allosteric modulators of Ste2p [41]. Both compounds enhanced the biological activity of α-factor, but did not compete with the α-factor binding to Ste2p. To determine if novobiocin and the 11-mer shared a common allosteric binding site, a biologically active analog of the 11-mer peptide ([Bio-DOPA]11-mer), where DOPA is l-3,4-dihydroxyphenylalanine, was chemically cross-linked to Ste2p in the presence and absence of novobiocin. Immunoblot probing for the Ste2p-[Bio-DOPA]11-mer complex revealed that novobiocin markedly decreased the cross-linking of the [Bio-DOPA]11-mer to the receptor, but the cross-linking of the α-factor analog [Bio-DOPA]13-mer, which interacts with the orthosteric binding site of the receptor, was minimally altered. This finding suggested that both novobiocin and [Bio-DOPA]11-mer compete for an allosteric binding site on the receptor. These results indicated that Ste2p provides a model system for studying allostery in a GPCR.

#### 3.3.2. Biased Agonists

**Figure 4:**
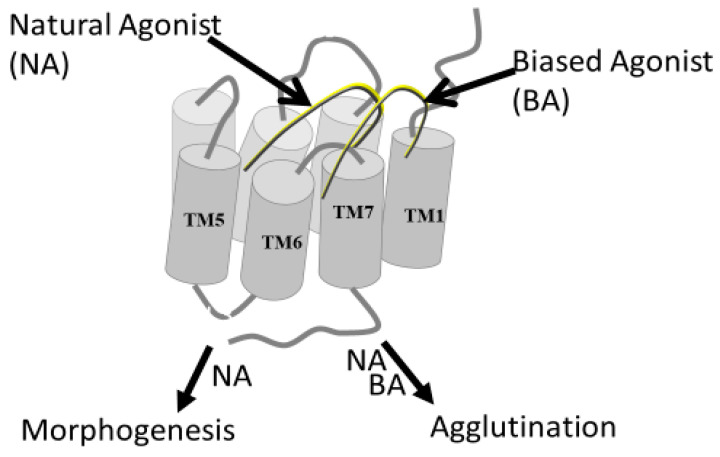
Biased agonism. This diagram shows two peptides, one the naturally occurring α-factor, a normal agonist (NA), and the other a biased agonist (BA) that preferentially initiates different signaling pathways (morphogenesis or agglutination). The BA initiates agglutination at a much lower concentration than that of NA, whereas the NA initiates both similarly [44].

GPCRs originally were thought to signal exclusively through a pathway initiated via the hetero trimeric G-proteins. However, it is now known that at least one additional pathway involving β-arrestin as a transducer also controls the response to certain ligands activating “GPCRs” [42]. A ligand that activates both the G-protein and β-arrestin pathways is termed a balanced agonist, whereas a biased agonist exclusively or preferentially activates one of these pathways [42,43]. During studies probing the structure-function relationships of various α-factor analogs, we determined the response of eight synthetic analogs of α-factor and measured two functional outputs of the mating pathway: morphogenesis (shmoo induction) and increased agglutinability, a pheromone-induced cellular property necessary for the mating process [44]. Most analogs induced increased agglutinability at lower concentrations than those at which they induced morphogenesis, and the ratio of the potencies for the two effects varied 140-fold among different analogs. Morphological response to pheromone required exposure for at least 90 min, but increased agglutinability followed exposures of 20 s. At that time, we hypothesized that the response of *S. cerevisiae* to α-factor may be mediated by more than one receptor. We now know that the *S. cerevisiae* genome encodes only one receptor for α-factor, and that this yeast possesses an arrestin-like protein that regulates pheromone response [45]. Thus, these studies appear to represent an early observation of biased agonism by showing that different ligands induced different physiological responses, perhaps one involving arrestin or an undiscovered alternative pathway. Figure 4 shows a diagrammatic representation of hypothetical biased agonism in yeast.

## 4. Identification of Ligand-Receptor Interaction by Cross-Linking Studies

Structure activity investigations coupled with site-directed mutagenesis are widely used to gain insights into peptide-GPCR contacts at the residue level. As outlined in Section 7.1, such studies provided support for the interaction of Gln^10^ of α-factor with residues 47 or 48 of Ste2p and showed that the N-terminal residues of the pheromone interacted with Y266. However, in order to gain more direct evidence for the positioning of α-factor into the active site of Ste2p, we employed crosslinking approaches. The use of chemically or photochemically activated groups to affinity label proteins is a concept that dates to the 1960s based on seminal studies by Singer [46,47] and Westheimer [48,49]. Many chemically reactive groups such as haloketones and bromoacetyl moieties result in nonspecific reactions and are only reactive with nucleophilic sidechains. In contrast, photoactive moieties such as azides and carbenes have a much greater range of reactivities. Nevertheless, the incorporation of these moieties into stable peptides is often challenging. In addition, many of these require photoactivation by light with wavelengths below 300 nm. Light below 300 nm can promote oxygen-mediated side reactions involving aromatic and sulfur-containing residues [50]. In contrast, the diphenylketone group developed by Prestwich [51] is easily incorporated into a peptide sequence as benzoyl-l-phenylalanine (BPA), resulting in stable products that can be stored until use and activated by 365 nm light. Therefore, in our photocrosslinking of α-factor and Ste2p, we choose to use BPA.

To understand the binding contacts between α-factor and Ste2p, we synthesized a BPA-scanned series of the tridecapeptide in which the photoactivatable moiety was sequentially inserted into every position except for Gly^9^, which was known to require either Gly or a d-residue. The crosslinking of the peptides was initially followed by tagging a Tyr residue with iodine-125 [52]. Earlier studies had shown that the iodination of Tyr^13^ of α-factor resulted in an inactive pheromone [53], but that this Tyr could be replaced with a variety of aromatic groups [54]. Therefore, we replaced Trp^3^ with Tyr and inserted phenylalanine in position 13 for these studies. Biological and binding assays allowed us to conclude that residues 1, 3, and 13 could be used for crosslinking analysis. After photoactivation and the confirmation of the specific insertion of the analogs into the Ste2p active site, the tagged receptor was fragmented chemically and enzymatically and the radioactive fragments were characterized using polyacrylamide electrophoresis (PAGE) [52]. These studies revealed that residue 1 interacted with a region of Ste2p involving residues in the extracellular end of TM6, the third extracellular loop (EL3), and the 7th TM helix (residues 251–294). Despite these exciting results, it proved challenging to work with radioactive iodine, and the use of this tag also required the replacement of the natural Tyr^13^ in the sequence. Moreover, using PAGE to assess contact points is, at best, a low-resolution approach, because fragments close in molecular weight are not always easily distinguishable, and because membrane peptides can move aberrantly on acrylamide gels. Our ultimate goal was to use mass spectrometric (MS) techniques to pinpoint the crosslink position. Consequently, we used biotin as the tag.

Biotin is easily inserted into peptides, is stable, and binds with a remarkably high affinity (K_d_ ≈ 1 × 10^−15^ M) to avidin. This allowed the detection of the crosslinked peptide using an avidin-horseradish peroxidase assay. In addition, background crosslinking can be minimized by using an avidin affinity column to fish out the covalent peptide-receptor that results from photocrosslinking. This approach proved to be highly efficient, and allowed us to identify the crosslinked region using MS and also to determine that while positions 1 and 3 of α-factor interacted with residues in the TM5-TM7 region of the receptor, BPA^13^ crosslinked to a residue in the F55-Arg58 sequence that was near the extracellular end of TM1 [55]. Based on these findings and the results of site-directed mutagenesis, it appeared that the 13-residue pheromone interacts with residues in the first transmembrane helix and spans the 7-helix bundle of the Ste2p receptor to react with residues in the transmembrane domains 5, 6, or 7 or in the intervening loops. The involvement of residues at both the amine and carboxyl terminus in crosslinking interactions with noncontiguous regions of Ste2p was consistent with what we knew about the role of these regions of the pheromone in binding. Later, we ascertained that the activation of the receptor involved these and other regions as well (see below).

**Figure 5:**
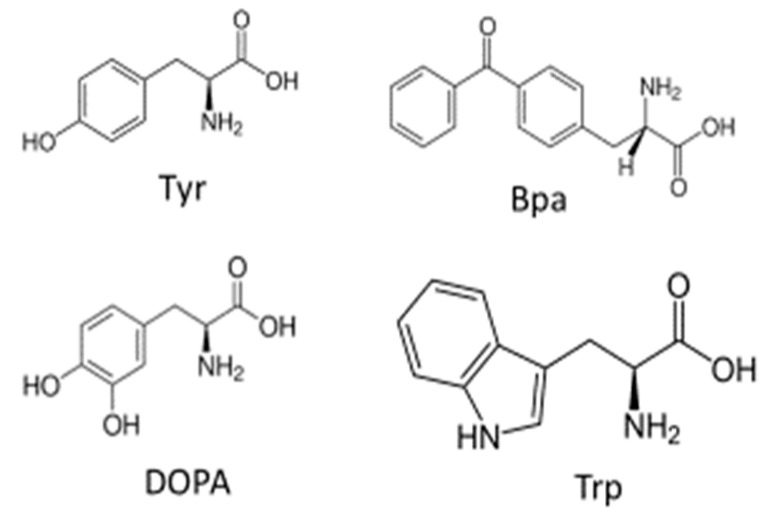
Structures of tyrosine (Tyr), 3,4-dihydoxyphenylanine (DOPA), tryptophan (Trp), and 4-benzoylphenylalanine (BPA).

One concern of crosslinking studies using peptides with amino acid derivatives is the possibility that they may interact with the receptor differently than the naturally occurring (or Wild-Type, WT) pheromone, thereby leading to erroneous conclusions. The diphenylketone moiety is large and clearly has different steric requirements than the phenolic group of Tyr^13^. It is also different chemically from Trp, however, the overall size of the side chains of BPA and Trp is similar (Figure 5). To gain evidence for the WT-like behavior of the analogs, a suite of bioassays, binding analyses, and competition experiments were conducted as controls as required for such analyses.

To test the influence of the BPA moiety on the binding of the α-factor analogs and the consequent conclusions of our crosslinking studies, we decided to use the dihydroxyphenyl moiety. This moiety was investigated by Kodadek and colleagues and was discovered to crosslink into nucleophilic centers upon activation with periodate [56,57]. The advantage of using dihydroxy-l-phenylalanine (DOPA) compared to BPA for crosslinking at Tyr residues is that Tyr and DOPA are similar in size. DOPA-containing peptides are also synthesized by standard solid-phase procedures and are quite stable in storage. In principle, a close analog of Tyr from the perspective of size would be azidophenylalanine, that may be generated from peptides containing aminophenylalanine. However, the azido analog is not stable and must be generated at the time of use, and the photoactivation of azidophenyl groups requires 254 nm light, which causes significant side reactions.

DOPA-containing α-factor analogs were synthesized to clarify the outstanding issues related to the contact of α-factor positions 1 and 13 with Ste2p. We found that upon periodate activation, DOPA^1^ and DOPA^13^ α-factor analogs were rapidly and selectively inserted into the receptor binding site [58,59]. The attachment of biotin to the Lys^7^ α-amine of the pheromone resulted in crosslinked fragments that could be isolated and enriched using avidin affinity chromatography. Most importantly, the DOPA^13^ α-factor crosslinked to Ste2p, Cys59, supporting our previous conclusion that BPA^13^ α-factor interacted with Arg58 of the receptor. Furthermore, using affinity purification and MS-MS approaches, we determined that DOPA^1^ α-factor, which had previously been found to crosslink to the 251–294 region using BPA^1^α-factor, was linked to Lys269. This was the first time that MS/MS was used to sequence and precisely identify the point of linkage of a peptide with its cognate GPCR [59].

In summary, affinity labeling approaches are now developed to the point that together with biotin tagging, detailed information concerning the residue-to-residue interactions between peptides and receptors can be obtained. By the use of agonists and antagonists, this method in principle can distinguish contacts that may be critical for activation. Many peptide hormones are highly flexible molecules. The α-factor, which is of undefined structure in solution, must undergo an induced fit process, as it binds to and catalyzes the transition of Ste2p from the inactive to the active state. This pathway must involve inherent flexibility in both the peptide ligand and the receptor. The biochemical approaches discussed in this section yield insights into this process even in the absence of a high-resolution structure for the receptor-ligand complex.

## 5. Insights into Ste2p Functional Assemblies from Fluorescence Approaches

The information summarized above from structure activity, crosslinking, circular dichroism, and nuclear magnetic resonance spectroscopy investigations indicated that the bound structure of the α-factor included a bend around a Pro-Gly turn in the middle of the peptide. Our working model hypothesized that the function of residues 5 to 9 of the peptide was primarily to allow the proper orientation of the activity determinant at the N-terminus and the binding determinant at the C- terminus of the pheromone. Therefore, we sought information about the environment of the Lys^7^ residue in the bound state by designing α-factor derivatives containing fluorescent tags. Initially, we worked with dansylated and fluoresceinated α-factor. In our hands, neither of these proved useful in studying the binding of the pheromone or the environment of the fluorophore. We note, however, that fluorescein attached to a Cys residue added to the C- terminus of α-factor was used successfully in a binding assay during the purification of Ste2p [60]. Ultimately, we chose the 7-nitrobenz-2-oxa-1,3-diazol-4-yl (NBD) fluorophore as our probe. This moiety is relatively small in size, can be excited at 480 nm, has a weak fluorescence at ~540 nm in polar environments, and has a very strong fluorescence which is blue-shifted in nonpolar environments. The difference between the NBD fluorescence under polar and nonpolar conditions makes this fluorophore very attractive for probing GPCR binding sites. Studies with Lys7(NBD)α-factor revealed that the NBD group was in a decidedly nonpolar environment [61]. We studied the interaction of the position 7 side chain with Ste2p further by synthesizing a series of α-factor analogs in which Lys^7^ was replaced with ornithine, 2,4-diaminobutyric acid, and 2,3-diaminopropionic acid, and then derivatizing the amine group in the sidechain with NBD. These analogs differed in the distance of the NBD group from the peptide backbone by one methylene group. What was striking was the finding that this systematic change led to a periodic variation in the polarity of the environment of the chromophore from nonpolar to polar and back. The accessibility of the NBD group in the side chain was also probed by fluorescence quenching studies [62].

The finding that the Lys7 side chain did not significantly affect the activity or affinity of α-factor [19] ultimately made the derivatization of this side chain ideal for probing the mechanism of activation of Ste2p. In our early studies, we worked almost exclusively with yeast membranes. As a precautionary note, the use of membranes can lead to changes in receptor-ligand interactions compared with the use of whole cells. Under in vivo conditions, GPCRs are found in cell membranes in the context of an intact cell. The use of isolated membranes and the possible changes in the composition of these supramolecular structures during their isolation can lead to changes in the receptor-ligand interactions compared to those found in whole cells. For example, it is known that the cholesterol gradients in membranes can affect the activity of adrenergic receptors [63]. In addition, membrane preparations often have significant nonspecific binding. This may have resulted in our inability to study the differences in the binding of α-factor agonists and antagonists. Ultimately, the availability of a variety of agonists and antagonists labelled with NBD at position 7 or 3 was a critical aspect of unraveling some of the subtle details of the α-factor-Ste2p interaction.

A chance meeting at a Gordon Conference in the late 1990s led to a series of discussions that resulted in a 20-year collaboration with a yeast geneticist who also had a very strong biophysical foundation. Working together with Mark Dumont led to some major developments in our studies of the α-factor/Ste2p paradigm. Mark solved the problem of background binding by employing fluorescent-activated cell sorting (FACS) in studies of the activation and binding of Ste2p. In these studies, whole yeast cells were used, and the nature of the cell-sorting instrumentation resulted in fluorescence measurements on a very small volume of cell suspension. The use of whole cells allowed the elimination of some of the nonspecific sticking by working with isogenic knock-out strains as a control, and the small volume minimized fluorescence from the solution. Together, this led to highly accurate binding constants, the ability to rapidly and conveniently compare environments for different ligands, and the development of screens for mutants with desired phenotypes.

**Figure 6:**
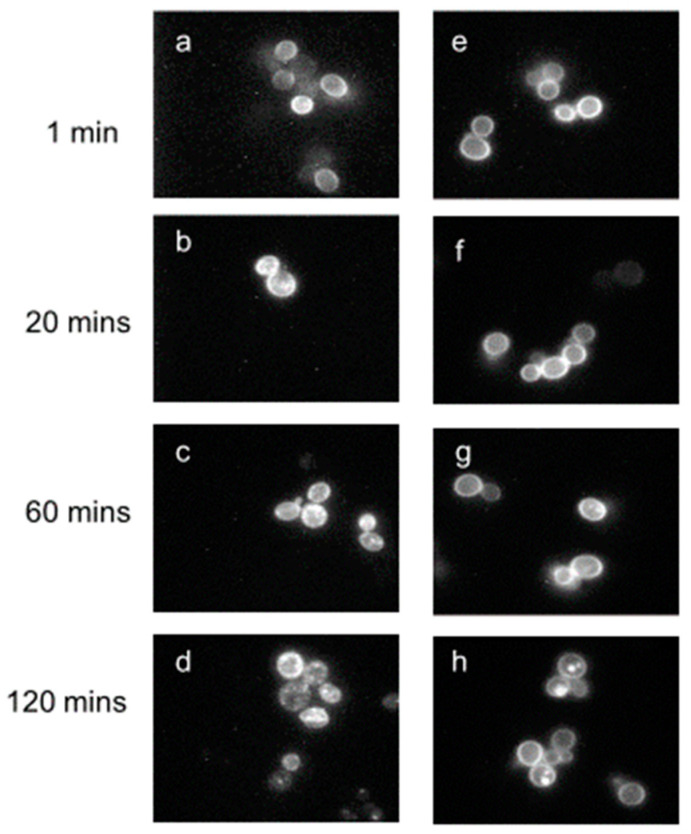
Endocytosis of [K7(NBD),Nle12]α-factor detected with fluorescence microscopy. Cells expressing full-length receptors (left panels) or truncated receptors (right panels) from multicopy plasmids were incubated with the fluorescent ligand at 30 °C for (**a**,**e**) 1, (**b**,**f**) 20, (**c**,**g**) 60, and (**d**,**h**) 120 min. The ranges of image intensities in panels (**e**–**h**) are approximately 2-fold greater than those for panels (**a**–**d**), indicative of the stronger fluorescence from the truncated receptors. Taken with permission from [70].

Previous investigations had provided evidence that Ste2p was removed from the yeast membrane by ligand-induced endocytosis [64,65,66], and that this removal was accompanied by the uptake of α-factor [67]. Fluorescence microscopy beautifully demonstrated the presence of the receptor at the plasma membrane and the time course of its disappearance from the membrane to subcellular locations using [Lys7(NBD)]α-factor (Figure 6). In order to eliminate any influence of endocytosis, all binding affinities were determined under conditions (0 °C and energy inhibitors or using carboxyl terminal-truncated receptors) where endocytosis was insignificant [68,69,70].

This system allowed a fast determination of optimal conditions for studying the binding of fluorescently labeled pheromones. Using flow cytometry, the binding of fluorescent α-factor to both full-length and wild-type receptors expressed by single or multicopy plasmids was subject to a detailed kinetic analysis. Details in terms of the binding curves and extraction of kinetic constants are provided in the literature. Binding was found to be best fit to a two-step process, presumably involving a change in the conformation of the pheromone and the concomitant change in the environment of the fluorophore [70]. This observation was consistent with previous conclusions based on the activity and binding affinities of various α-factor analogs. Most important, this work clearly demonstrated the advantage of fluorescence-activated cell sorting for a careful kinetic analysis of peptide-GPCR interactions.

The utility of the FACS approach was clearly demonstrated in a detailed analysis carried out in collaboration with the Dumont laboratory that used fluorescently labeled agonists and antagonists to screen for mutations that changed the emission spectra of the bound ligands [71]. The goals of the screens were to discover mutations that caused a change in the environment of the NBD probe on the position 7 side chain in an α-factor agonist or antagonist. The basic strategy of our approach was based on the observation that NBD in [Lys^7^(NBD)]α-factor (a strong agonist) was in a non-polar environment (blue shifted) when it was bound to wild-type Ste2p. Mutations in Ste2p that resulted in a red shift in the fluorescence of the agonist but which retained a strong binding and biological activity could be interpreted as due to residues that directly interacted with the pheromone. Similarly, for antagonistic peptides such as [d-Tyr^3^,Lys^7^(NBD)]α-factor or desTrp1,desHis^2^[Lys^7^(NBD)]α-factor, the NBD fluorescence indicated a less polar environment (red shifted), and the screen identified receptor mutations that resulted in the antagonists gaining signaling activity.

**Figure 7:**
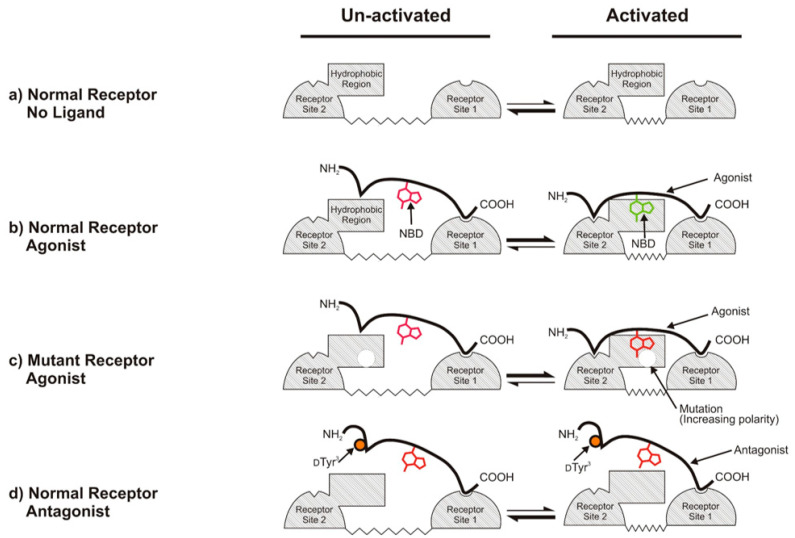
Model for the binding and activation of normal and mutant α-factor receptors by different ligands. Ligand-interacting surfaces on the receptor are indicated by cross-hatching. Site I interacts with the C-termini of ligands and is likely to consist of the extracellular end of the first transmembrane segment. Site 2 interacts with the N-terminus of ligands and most likely consists of the extracellular end of the sixth transmembrane segment and the third extracellular loop of the receptor. The conformational equilibrium between the inactive and activated states of the receptor is represented as a change in the distance between Site 1 and Site 2. (**a**) In the absence of ligand, the receptor favors the inactivated state with a large separation between ligand contact sites. (**b**) Binding of the agonist to normal receptors favors the activated state. (**c**) Mutations affecting the emission spectrum of bound agonist change the environment of the fluorophore (red shift) without altering the receptor ligand binding or, generally, activation. (**d**) Binding of ligands that act as antagonists toward normal receptors (labeled antagonist) does not alter the conformational equilibrium of the receptor. Activation-specific interactions with the N-terminus of the ligand are blocked by the d-Tyr^3^ substitution (orange circle), leaving the NBD group attached at Lys^7^ exposed to the solvent, and the C-terminal region of the antagonist (which is identical to the corresponding region of agonist) maintains a high-affinity interaction with the receptor. Adapted with permission from [71].

Combined with random mutagenesis, the screen rapidly identified a spectrum of mutations that changed the environment of the fluorophore. The screen benefitted from the fact that only mutations that are compatible with plasma membrane expression are identified, thus eliminating issues due to differential expression of mutations. By combining fluorescence characteristics with the binding affinity and signaling efficiency, mutations that directly and indirectly affect the environment were identified. The output of this screen was very rich and identified specific amino acids that appear to interact with the NBD in agonists. Virtually all of these amino acids were at the extracellular ends of transmembrane domains or in extracellular loops. None of these mutations led to significant changes in ligand affinity or signal transduction. In contrast, the environment of the NBD fluorophore in antagonistic peptides, which is on the same Lys^7^ side chain, was affected by mutations that appeared to cause changes in the receptor conformation and were often associated with a constitutive signaling phenotype. The results were used to develop a mechanism for the binding/activation pathway of the α-factor-Ste2p pair that differentiated between agonists and antagonists (Figure 7).

Perhaps as important as the insights into details of receptor ligand interactions in the yeast mating system was the development of a powerful system that can be used to probe structural changes and residue-to-residue interactions between a peptide ligand and its cognate GPCR. Since mammalian GPCRs can be expressed in yeast and since fluorophores are readily attached to many peptides, this approach should complement other mutagenesis methods in the examination of receptors that cannot be or have not been crystallized.

## 6. Studies of Ste2p Fragments: A Role for Peptide Synthesis and NMR Studies

In the 1980s and 1990s, very little progress was made on the crystallization of membrane proteins. Indeed, at that time the gold-standard model for the structure of a heptahelical membrane protein was based on an electron microscopy study of bacteriorhodopsin [72,73,74], a proton pump from purple membranes of *Halobacterium salinarum*. During those years, several laboratories attempted to study the structure of GPCRs using what was called a reductionist, or divide and conquer, approach, whereby the specific domains of the receptor were prepared and their structural tendencies and interactions with membranes were examined. The justification for such studies was based on the observation that split membrane proteins could reassemble to form functional transporters, pumps, and receptors [75,76,77,78]. Particularly relevant to our interests, regions of Ste2p could be expressed in S. cerevisiae and the noncovalent reassembled receptor could signal in response to α-factor [79]. This latter study showed that even a single transmembrane domain (TM) could reassemble with the complementary six TM Ste2p fragment to form a functional receptor. Thus, we hypothesized that we could learn about aspects of the structure of Ste2p by studying fragments corresponding to specific regions of the receptor. Accordingly, in the early 1990s we began investigations on the biophysical propensities of fragments of the α-factor receptor [80]. These studies required the development of synthetic and biosynthetic approaches to prepare various Ste2p domains. Much of this work has been previously summarized [81,82,83] and will not be discussed in detail. In the following section, we will discuss primarily what we learned by studies of large fragments of a GPCR, and how this work may have contributed to understanding the structure of the intact receptor.

Initial studies from our laboratory involved the chemical synthesis of peptide surrogates for the loop and single transmembrane regions of Ste2p and biophysical investigations on these peptides using CD spectroscopy [80]. Later, these studies were extended using 1D and 2D NMR spectroscopy to provide structural details at the residue level [84,85,86] and Attenuated Total Reflectance-Fourier Transform Infrared Spectroscopy (ATR-FTIR) to study the tilt angle of single transmembrane domains in bilayers [87]. Significant challenges encountered with these peptides were the result of the poor solubility of the fragments and their tendency to aggregate during synthesis, purification, and sample preparation. We also learned that the seven individual TMs of Ste2p, while assuming helices in peptide mimetic environments, had unique biophysical tendencies [85,87]. Similar conclusions were reached by other laboratories on peptides corresponding to the TM regions of bacteriorhodopsin [88] and rhodopsin [89]. Surprisingly, peptides corresponding to the 6th transmembrane of Ste2p formed a kinked helix in organic aqueous membrane mimetics but assumed β-like structures in sodium dodecyl sulfate (SDS) micelles [85,87]. These results provided experimental support that context, in particular interhelical interactions, was critical in the determination of the structures of regions in the intact receptor.

The findings of investigations on single TM peptide surrogates led us to study the multidomain regions of Ste2p, including those containing a helix linked to the cytosolic terminus, and those containing two and three TM helices connected by intervening loops. The chemical synthesis of large peptides as surrogates for fragments of a GPCR is extremely difficult. The highly hydrophobic TM domains composed of many β-branched amino acids often form intermolecular structures on resins, leading to chain termination and the failure of the synthesis. The successful use of native chemical ligation has not been widely reported for membrane proteins, with some notable exceptions such as the potassium transporter [90,91,92]. Moreover, given the helical nature of the GPCR domains and the poor chemical shift dispersion associated with helical proteins, it is critical to have uniformly isotopically labelled peptide surrogates for high-resolution NMR studies. Assembling isotopically labeled proteins through chemical synthesis is economically unfeasible, although semisynthesis using both expressed and chemically assembled peptides can lead to segments of a protein that are “invisible” in the NMR spectrum. To prepare large fragments of Ste2p, we developed biosynthetic methods to express and purify polypeptides containing one, two, and three domains that could be studied by multidimensional heteronuclear NMR. Our biosynthetic approach was modelled on work from the Opella laboratory that used TrpΔLE-fusions, a 27-kDa protein used to direct the expression of fusion proteins into inclusion bodies [93,94]. The inclusion bodies were then isolated, and after purification the membrane protein was released by cyanogen bromide cleavage. This method proved highly efficient; >100 mg of fusion protein could be isolated per liter of culture medium, and we had no difficulty preparing the tens of mgs of the final peptides used in our NMR studies. In later evolutions of our studies, we optimized approaches to directly express regions of Ste2p. This eliminated the need to cleave the product from the fusion protein, and was especially useful in preparing peptides with Cys at selected positions for spin-labeling [95]. Zerbe and co-workers have developed a complete set of approaches for the biosynthesis of neuropeptide Y subtype 4 receptor (NY4) fragments [96]. The experience with the synthetic single TM peptides was extremely valuable in optimizing conditions for the isolation of [^15^N], [^15^N,^13^C], and [^15^N,^13^C,^2^H]-labeled proteins, as well as proteins isotopically labeled at specific amino acid residues [97].

In 2004, we met Oliver Zerbe, an NMR spectroscopist from the University of Zurich, and began a fruitful study on the conformations assumed by the multi-domain Ste2p surrogate peptides in micelles. One such surrogate peptide, EL3-TM7-CT40, contained residues corresponding to the third extracellular loop, the seventh transmembrane helix, and the carboxyl terminus of the Ste2p receptor (see Figure 2). In contrast to CD and fluorescence spectroscopy, NMR is usually carried out in the 100 µM to mM concentration range. To achieve such concentrations in aqueous media, many laboratories placed charged residues, specifically oligolysines, at the termini to increase solubility [88,98,99]. This is a trick that goes back to the 1960s and relies on the belief that residues at the end of a polypeptide domain will not influence its structure [100]. However, we also wanted to interrogate the TM domains of Ste2p in the context of their contiguous loop and tail regions. We reasoned that the hydrophilicity of the residues in the loop and tails of a GPCR would help solubilize the TMs to which they were attached. Our first target was a construct containing TM7 of Ste2p attached to most of the third extracellular loop (EL3) on the amine terminus and 40 residues of the cytosolic tail at the carboxyl end. A detailed NMR study of this 72-residue peptide showed that the different domains assumed distinct structures: TM7 formed a helix that was rather flexible around the Pro residue in dodecylphosphocholine (DPC) micelles, and the cytosolic carboxyl terminus (CT40) was excluded from the micelle core and assumed a mostly disordered structure [101]. Interestingly, the NMR analysis revealed that a region of CT40 had helical tendencies and interacted with the surface of the micelle. This is the only evidence that Ste2p has an 8th helix similar to the helix discovered in the crystal structure of rhodopsin [102]. This latter helix is thought to act as a conformational switch in this light-activated receptor [103], and in general the cytoplasmic terminus is involved in the regulation of GPCRs [104,105,106]. We subsequently carried out studies on mutations of Ste2p’s C-terminus (CT), described below, that showed the contributions of the C-terminus to various Ste2p functions [107].

The experience gained in the study of this multi-domain polypeptide taught us about critical factors that needed to be optimized to achieve high-quality NMR spectra for fragments of Ste2p in membranelike media. Key issues were the choice of detergent, the lipid/peptide ratio, the peptide concentration, and the temperature. We were also interested in understanding whether the membrane environment could be simulated by organic-aqueous media, and whether NMR assignments on short fragments could be transferred to or at least aid in the assignment of longer fragments. The helix formed by TM7 in the EL3-TM7-CT40 (a peptide containing part of the third extracellular loop, the entire seventh transmembrane helix, and 40 residues of the carboxyl terminus of Ste2p) construct was transient and showed high flexibility around the Pro residue in the middle of this region. TM1 also was also somewhat more flexible around a GXXXG sequence in the middle of this helix. This sequence has been shown to be involved in the formation of GPCR dimers in Ste2p [108] and other GPCRs and transmembrane proteins [109]. Often, TMs of GPCRs have polar and, less frequently, charged residues. The energy for inserting these polar and charged residues into a bilayer is unfavorable, and the intact receptor can compensate for this high-energy state by forming helix–helix interactions that satisfy the H-bonding needs of these polar side chains. This led us to explore whether Ste2p constructs with Ste2p TM1 (G31-T78), Ste2p TM1-TM2 (G31-T110), Ste2p TM1-TM2-TM3 (G31-R161), and Ste2p TM1-TM2-TM7 (G31-T114,T274-L340) (TM7 packs against TM1 in bundles formed by full-length GPCRs [110,111]) would form stable tertiary structures and integrate well into micelles. The availability of these constructs allowed us to conduct systematic analyses that provided answers to the above questions.

High-resolution NMR studies on membrane proteins must be carried out in an appropriate membrane mimetic environment. The ideal mimetic is the cell membrane itself. Obviously, direct studies on receptors in intact cell membranes would be compromised by the heterogeneous composition of lipids, sterols, and proteins in cell membranes. In addition, even in simplified, reconstituted membranes severe broadening will occur due to the slow tumbling times experienced with these high molecular complexes. Moreover, the structures formed by GPCRs and fragments of these molecules are dynamic, and some regions are in the intermediate exchange regime, also leading to peak broadening. Thus, due to these considerations solution NMR on GPCRs in liposomal (bilayer) mimetics is not possible. Such studies in simplified membrane systems are now the province of solid-state NMR investigations [112]. Due to this limitation, most NMR studies on membrane proteins and membrane peptides have been conducted in detergent micelles and organic aqueous mixtures. The historic record of the use of fluorinated organic solvents to study hydrophobic peptides by CD and NMR began in the 1960s [113], when trifluoroethanol (TFE) was found to support helical structures in linear oligopeptides. Subsequently, TFE and TFE/water mixtures were extensively used to study a diverse set of hydrophobic and amphiphilic peptides. Although TFE/water is now widely considered to be a structure-inducing solvent, we have found that careful control of the TFE/water ratios and monitoring by CD can lead to meaningful insights. Accordingly, we have carried out an analysis of Ste2p TM1-TM2 (G31-T110) and Ste2p TM1-TM2-TM3 (G31–R161) in both TFE/water mixtures and in detergent micelles.

In working with membrane peptides in detergents, it is essential that each micelle contains only one peptide molecule. We found that extremely large molar ratios of detergent to peptide were necessary to obtain resolved NMR spectra. At a 20:1 ratio, the [^15^N-^1^H] HSQC (heteronuclear single quantum correlation) spectra were extremely broad and peak duplication was observed. At ratios of 400:1 and higher, relatively narrow crosspeaks were obtained [114]. Similar observations of detergent/protein ratios had been made for a number of membrane proteins, and we are grateful to Stan Opella for calling this to our attention [115]. We have employed such high ratios in all analyses of multi-TM Ste2p fragments. The method to prepare the sample and the choice of detergent used in the analysis is also a critical variable [116,117,118] in conducting an NMR study. Many GPCR fragments are unique in their biophysical properties, and the detergent chosen and the conditions used for sample preparation need to be carefully optimized. SDS, which has previously been widely used as a membrane mimetic, should probably be avoided because of its high tendency to denature the membrane protein. As a corollary, researchers in this field are encouraged to give the details of sample preparation to enable colleagues to conduct complementary studies on their systems.

**Figure 8:**
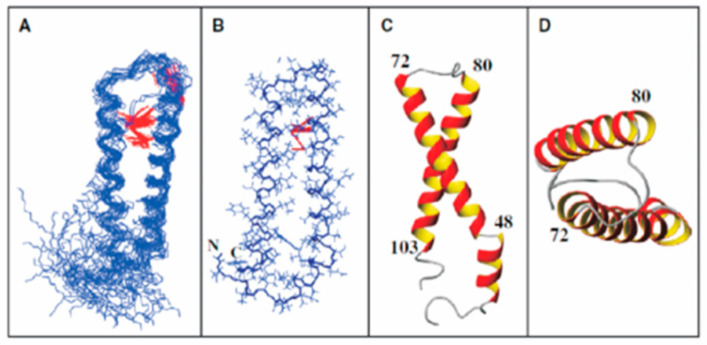
(**A**) Backbone representation of the ensemble of the 20 lowest energy conformers of Ste2p (G31-T110) superimposed over backbone atoms in the TM1-TM2 region comprising residues 39–103. Observed long-range NOE contacts are highlighted in red. (**B**) A single conformer from the ensemble additionally displaying the side chains. (**C**) Structure of a single conformer—view from the side of the membrane interior. (**D**) The same as **C**, but viewed from the cytoplasmic side. Taken with permission from [121].

Our side-by-side analysis of Ste2pTM1-TM2 (G31-T110), Ste2p TM1-TM2-TM3 (G31–R161), and Ste2p TM1-TM2-TM7 (G31-T114, T274-L340) led to some important conclusions [95,119,120,121]: (1) NMR was readily applied to study the structure of these membrane polypeptides, which contained from 80 to 181 residues and two or three transmembrane domains. (2) High-quality spectra were obtained in both TFE/water and micelles, although different detergents were found to give the best results for each peptide. (3) The NMR parameters obtained both in TFE/water and detergent micelles distinguished the putative transmembrane regions from loops and tail residues. Some variability was observed at the domain boundaries, and in general the helical regions found in the amine terminal tail and EL1 (Extracellular Loop 1, see Figure 2) were extended in TFE/water compared with the detergent. (4) Evidence was found for the formation of a metastable helical hairpin for Ste2pTM1-TM2 (G31-T110) in micelles (Figure 8), which also persisted in Ste2pTM1-TM2-TM3 (G31–R161) and Ste2pTM1-TM2-TM7 (G31-T114, T274-L340), but tertiary contacts involving TM3 and TM7 were not observed. (5) The packing of TM1 against TM2 resulted in the stabilization of the secondary structure of TM1. (6) A very high percentage (92–97%) of backbone atom assignments were achieved in both TFE/water and detergent. (7) Assignments could not be transferred from TFE/water to the detergent micelle system. (8) Assignments on smaller fragments—for example, Ste2p TM1-TM2 (G31-T110)—were very useful in making assignments of the same regions in a larger construct—for example, Ste2p TM1-TM2-TM7 (G31-T114, T274-L340). Whether these assignments can be transferred to the intact receptor is still being investigated (Oliver Zerbe, personal communication). The conclusions reached above on Ste2p and those by Zerbe and coworkers on fragments of the NY4 receptor [96,122,123,124] provide guidance for future studies on fragments of membrane proteins. Zerbe and coworkers have provided a detailed discussion on many of these issues in an excellent review of this field, and we have tried to not rehash those insights [124].

Should one undertake major efforts to study peptide surrogates to learn about the conformations assumed by regions of GPCRs? It is clear that studies on fragments of GPCRs reveal important information on the secondary structure of the different sequences in a GPCR. The ability of NMR studies to provide information about transient helices such as the amphiphilic helices in the N-terminus of Ste2p and NY4 and the 8th helix in the CT (C-terminus) of Ste2p is important and can help explain the role of these regions in ligand recognition, receptor regulation, and signal transduction. To accurately pinpoint these secondary structures, we believe that NMR studies should be conducted in environments that closely mimic the membrane. Although information on secondary structures can be derived in organic aqueous mimetics, there likely will be differences at the boundaries. Detergent with different chain lengths may also affect the helical boundaries in a receptor fragment. Indeed, this may also be true with the intact receptor. Investigations on receptor fragments are less likely to provide information on the tertiary structures assumed by many domains. Our analysis of the 3TM containing fragments of Ste2p and Zerbe’s studies on 2TM and 3TM containing fragments of NY4 suggest that, in most cases, these do not form tightly packed structures in micelles. Given the current state of the art, rather than studying GPCR fragments, it would appear more profitable to spend one’s efforts on studies of the intact receptor. In contrast to their use in structural evaluations, studies on fragments can provide valuable biophysical data on the proclivity of various TMs of a membrane protein to insert into a membrane. Comparison of the NMR parameters of Ste2p TM1 (G31-T78), Ste2p TM1-TM2 (G31-T110), and Ste2p TM1-TM2-TM3 (G31-R161) in the presence of micelles, along with membrane topology studies, suggests that TM1 of Ste2p will not integrate into the membrane until TM2 is present. This suggests a pathway for the folding of the receptor during biosynthesis.

Have the NMR studies of Step contributed to our understanding of the biochemistry of this GPCR? At the time of writing, there is still no crystal structure of Ste2p. Indeed, there is no structure for any member of the α-factor receptor subfamily. This is not due to lack of effort, as we are aware of major attempts in several laboratories to crystallize Ste2p. Thus, the structural information on this receptor only comes from the studies on fragments of the receptor. These studies have revealed that the GVRC(S)G (a GXXXG motif) present in TM1 is a relatively flexible motif. This flexibility would be consistent with the fact that the carboxyl terminus of α-factor can crosslink to residues in the GXXXG motif. If the TM1-helix were tightly packed in the 7 TM bundle, ligand binding might be energetically prohibitive. The presence of an amphiphilic helix in the N-terminus of both Ste2p and NY4 receptors, and the binding of this amphiphilic helix in NY4 with its ligand, provides a nexus for the interaction of ligands first with the membrane lipid, then with the N-terminus, and finally with the receptor’s ligand binding site [124]. Finally, the structural information on the N-terminus and the EL1 have been useful in guiding biochemical studies that have revealed the involvement of these regions of Ste2p in receptor activation and signal transduction.

## 7. Genetic Manipulation of the Receptor Structure

An advantage of using *S. cerevisiae* is the ability to utilize genetic engineering to quickly create mutations in the sequence of any given protein and the subsequent expression of a mutant form of the protein in its native yeast environment. Expression is followed by assays of activity, including response to various ligand variations, thus providing a powerful experimental system. Such genetic manipulations and facile cellular expression, were not available for mammalian GPCRs until relatively recently. Thus, we took advantage of the awesome power of yeast genetics to probe receptor structure and function. Once the sequence of *STE2* was determined [12], mutation, expression, and functional studies of mutant forms of the gene could be performed in a matter of weeks.

As stated earlier in this review, the cloning of *STE2* and analysis of its encoded protein showed that it was a 7-transmembrane protein of 431 amino acids [12]. The comparison of the structure of Ste2p to the cloned β-adrenergic receptor [14], biochemical evidence for α-factor binding to Ste2p [9,10,11], the discovery of *S. cerevisiae* genes encoding the beta and gamma components of a G protein involved in signal transduction [125], and the establishment of the coupling of the Gα protein to Ste2p for signaling [126] showed conclusively that Ste2p was indeed a peptide-activated GPCR.

Studies by Blumer and Thorner provided unequivocal evidence that the in vitro coupling of ligand and Ste2p required all components of the heterotrimeric G protein (Gα, Gβ, and Gγ subunits) [126]. Using isolated membranes at pH 8, they observed that high-affinity binding (K_d_ = 17 nM) occurred in the absence of a GTP analog, whereas low-affinity binding (K_d_ > 150 nM) was observed in the presence of 100 µM of a nonhydrolyzeable GTP analog. In contrast, our studies showed that, under in vivo conditions using whole cells and pH 5.8, the effect of the presence of Gα was dependent on the receptor (full-length or truncated) and whether the receptor was expressed from a multicopy or CEN plasmid [70]. Under these conditions, the full-length receptor showed a two-fold increase or no change in affinity, respectively. The truncated receptor showed about an eight-fold increase in affinity. The differences between our results and those of Blumer and Thorner might reflect the fact that we used whole cells at pH~6, whereas they used membranes at pH 8. They were unable to observe a GTP influence on the α-factor-Ste2p interaction at pH 6.0 [126]. Based on these results, it is not clear to us whether coupling with G protein has a significant effect on the affinity of α-factor for Ste2p.

As described in Section 3.2, we initiated studies to determine the structure of α-factor in the binding site of Ste2p. The underlying premise was that an understanding of the structure of the “key” (α-factor) that fit the “lock” (Ste2p) would lead to a mechanism of how a peptide hormone/pheromone triggered a signal transduction system. Studies using analogs of α-factor and affinity labeling gave us some idea of the residues of Ste2p that might be involved in binding. In parallel, we conducted genetic engineering of *STE2* in order to determine which Ste2p residues were involved in ligand binding.

### 7.1. Identification of Specific Residues Involved in Ligand Binding via Receptor Mutagenesis

Studies in other labs on Ste2p mutants with a linker insertion [127] or single-residue substitutions [128,129,130] and on receptor chimeras between *S. cerevisiae* and *S. kluyveri* Ste2ps [131,132] indicated that the α-factor binding site might include determinants contributed by several transmembrane segments and extracellular loops. We used a combination of Ste2p mutations and α-factor analogs to probe for the receptor binding site.

Random mutations in Ste2p were generated and were screened for variants that responded to antagonists [19]. Multiple mutations were detected in each mutant receptor recovered from the screen; then, site-directed mutagenesis was used to create single-site mutant receptors. Three receptors containing the mutations F55V, S219P, and S259P were analyzed for their biological responses to various α-factor analogs and for their ligand binding profiles. Cells expressing each of the mutant receptors responded to α-factor as well as or better than wild-type cells in a growth arrest assay. In contrast, the binding of α-factor to the F55V and S219P mutant receptors was at least 10-fold reduced in comparison to wild-type receptor, indicating a complex non-linear correlation between binding affinity and biological activity. This is likely due to the fact that the activation of a small number of receptors can still result in a maximal biological response [133]. Cells expressing the above mutant receptors responded to some normally inactive α-factor analogs in biological assays, despite the fact that these analogs had a low affinity for Ste2p, as measured by binding assays using whole cells. The analysis of these mutant receptors confirmed previous findings that the first and sixth transmembrane regions of Ste2p are important for ligand interaction, ligand specificity, and/or receptor activation to initiate the signal transduction pathway. A key outcome was that, consistent with our affinity labeling and structural studies, residue F55 of Ste2p, which is contiguous with the GxxxG motif (GVRSG in Ste2p in TM1), was involved in both ligand binding and signal transduction.

Our photoaffinity labeling studies [52] led us to test a hypothesis that the extracellular portion of TM6 of Ste2p interacts with the N-terminus of α-factor. We mutated nine residues to alanine in a portion of TM6 adjoining EL3 adjacent to the membrane extracellular interface [134] adjoining EL3. The only mutant receptor that exhibited changes in binding or receptor function was Ste2p (Y266A), which did not signal and had a five-fold decrease in α-factor affinity. We concluded that Y266 is a strategic residue, critical for receptor function and for the recognition of the N-terminus of α-factor. In contrast to Ala substitution, Phe or Trp substitution at Y266 retained receptor function, suggesting that aromaticity at this position was critical. A partial tryptic digest revealed that, in the presence of α-factor, a different digestion profile for Y266A receptor was generated in comparison to that for WT receptor. The difference in trypsin-sensitive sites and their negative dominance indicated that the Y266A receptor was not able to switch into an “activated” conformation upon ligand binding. In comparison to WT Ste2p, Ste2p (Y266A) showed an increased binding affinity for N-terminal, alanine-substituted α-factor (residues 1–4), and the antagonist [desW^1^, desH^2^-α-factor. The results suggest that Y266 is part of the binding pocket that recognizes the N-terminal portion of α-factor and is involved in the transformation of Ste2p into an activated state upon agonist binding.

The studies on chimeras of the *S. cerevisiae* and *Saccharomyces kluyveri* α-factor receptors [131,132] identified small extracellular regions that determined the specificity of ligand binding and/or receptor activation. In *S. cerevisiae*, these regions corresponded to residues 47–49 (STV) at the junction between the N-terminal domain and TM1. In light of these chimera studies and the turn structure in residues 7–10 of α-factor that we had found by structural analysis of the ligand, we hypothesized that the 10th residue of α-factor interacted with residues 47–48 of the receptor. To test this hypothesis, we constructed receptor mutations and tested the binding affinities and biological activities of these constructs with various α-factor analogs [135]. Mutant receptors differed in binding affinity and potency for signal transduction measured by a gene induction assay. One mutant receptor (S47K, T48K) had dramatically reduced affinity and activity for [Lys^10^]- and [Orn^10^]α-factor. In contrast, the affinity of these two analogs was increased greatly in a S47E, T48E mutant receptor. These results demonstrated that, when bound to its receptor, the 10th residue (Gln) of the *S. cerevisiae* α-factor is proximal to the Ser47 and Thr48 residues in the receptor and is involved in ligand binding.

We explored a very different approach to identify receptor residues involved in ligand binding site(s) by incorporating the photoactive BPA residue into Ste2p [136] using a method developed in the Schultz lab called unnatural amino acid replacement (UAAR) [137,138]. Yeast harboring a cognate tRNA/aminoacyltRNA synthetase pair specifically evolved to incorporate BPA, in response to an amber codon replacing an amino acid codon in *STE2,* allowing the biosynthesis of BPA-substituted Ste2p in its native cell. Several of the expressed BPA-substituted Ste2p receptors (F55 located in TM1 and Y193 located in EL2) exhibited high-affinity ligand binding and were biologically active, as measured by the growth arrest of whole cells in response to α-factor. These two BPA-Ste2p mutants were able to selectively capture α-factor in the putative ligand-binding site after photoactivation, providing direct evidence for the contact of these side chains and bound α-factor. To our knowledge, this was the first experimental evidence documenting an unnatural amino acid replacement in a GPCR expressed in its native environment, and the use of a mutated receptor to photocapture a peptide ligand. Similar UAAR experiments were carried out subsequently on other GPCRs [139,140,141,142].

The crystallization of many eukaryotic GPCRs is still limited by the thermal instability of these receptors in mixed micelles. In a collaboration with the lab of Mark Dumont, we pursued a strategy to identify thermally stabilized variants of Ste2p [143]. This project was initiated in order to find a mutant that might be more amenable to crystallization as attempts to crystallize the naturally occurring Ste2p had failed despite the attempts of several labs. A battery of destabilized temperature-sensitive variants was isolated based on the loss of signaling function and decreased levels of binding of a fluorescent ligand. These single amino acid substitution mutations were located at the extracellular face of transmembrane domains 1, 2, 5, and 6; the N-terminus; and the 1st extracellular domain (EL1). We then screened randomly mutagenized libraries of clones expressing these temperature-sensitive variants for second-site suppressors that restored elevated levels of binding sites for the fluorescent ligand. Seven mutations (R58G, R58S, F119L, F119S, M218T, L248R, and T279A) provided global suppression, in that they were capable of suppressing more than one of the tested temperature-sensitive starting alleles. The R58 mutation on the predicted second transmembrane helix was unique among the suppressor substitutions in its ability to provide the substantial suppression of the temperature-sensitive phenotype of the S95Y substitution, and only when R58 was replaced with amino acids with small side chains. R58 has been mentioned above as a residue of Ste2p involved in ligand binding. This method did not result in a more stable Ste2p that was crystallizable. Although great progress in finding thermally stabilized GPCR mutants has been made both computationally [144] and experimentally [145], the process is often quite cumbersome and takes even an experienced laboratory a year to succeed. One reason for the long time is the need to perform targeted site-directed mutagenesis of preselected amino acid residues. Although the fluorescent screen described above has not, to date, resulted in a crystallizable Ste2p (Mark Dumont, personal communication), the use of a fluorescence-activated cell sorter (FACS) screen and random mutagenesis, in principle, could be applied to mammalian GPCRs.

Although GPCRs including Ste2p contain several distinct domains often roughly divided into the extracellular N-terminus, transmembrane bundle, and intracellular C-terminus [11], the majority of studies on GPCR structure and function have focused on the seven transmembrane helices. The focus on the TMs appears to be due their role as the major binding region of small molecule ligands and the conformational changes they undergo upon ligand binding to initiate the signal transduction cascade. However, a number of studies indicates that the N-terminus also plays an important role in receptor function [146,147,148]. We decided to interrogate the N-terminus of Ste2p by determining the consequences of deletions and mutations in the N-terminus on Ste2p function. The experiments outlined below led to a number of unexpected findings: the N-terminus had structural sub-domains, it was involved in Ste2p dimer formation, it interacted with specific sub-domains of the EL1 domain, and it played a role in Ste2p conformational change upon ligand binding.

### 7.2. Function and Structure Determination of Ste2p by Mutagenesis

#### 7.2.1. Exploring the Extracellular Region of Ste2p: The N-terminus and EL1

The N-terminus of GPCRs has not been probed extensively for its structure and function in comparison to transmembrane domains [148]. This is due in part to the intrinsically unstructured N-terminal domain and the consequent lack of inclusion of N-termini in crystal structures. In 2007, the lab of Michelle Loewen showed, by deletion of the first 20 amino acids of the N-terminus, that this portion of Ste2p was implicated in mating efficiency but not growth arrest. A later study from the Loewen lab concluded that cell-cell interaction between the N-termini of pheromone receptors of *MAT**a*** and *MATα* cells was necessary for mating [149]. Previous mutations of Ste2p had shown that two glycosylation sites in the N-terminus were not required for mating or the initiation of signaling by α-factor [150], and that the N-terminus alone does not act as a canonical signal peptide, as shown by topology studies [151]. Intrigued by these findings, we carried out a systematic deletion of regions of the N-terminus and measured the consequences of these changes on Ste2p expression and signaling.

The deletion of residues two through 10 of Ste2p resulted in an overexpression of the receptor on the cell surface, thus implying that this region, or its influence on the conformation of the receptor, acted to negatively regulate receptor expression [152]. In addition, we had shown previously that residues 20 through 30 formed a β-sheet [153], and that residues in extracellular loop one (EL1) formed a 3_10_ helix [154]. We hypothesized that the N-terminus and EL1 interacted during activation of the signal transduction pathway. To test this hypothesis, we created single and double cysteine mutants and measured their interaction by cross-linking [155]. Such mutants showed direct interactions between the N-terminus and EL1 and, in addition, showed that the N-terminus was involved in formation of Ste2p-Ste2p dimers.

A complete scan of EL1 was achieved by replacing all of its residues one-by-one with cysteine [156]. A number of these mutants retained the ability to bind α-factor but exhibited reduced signaling. In particular. L102C, N105C, S108C, Y111C, and T114C signaled poorly or not at all, implying that certain residues in EL1 were involved in a switch between the resting and active receptor conformational states. The periodicity of this phenotype was consistent with the presence of a 3_10_ helix in this region of EL1. This conclusion was confirmed by modeling and circular dichroism analysis on an EL1 peptide, which also suggested that residues 126 to 135 formed two short beta-strands [154]. The putative involvement of EL1 in the conformational switch that activates the Ste2p receptor upon α-factor binding prompted biochemical studies to probe environmental changes of specific residues in this loop.

The systematic mutation of EL1 residues to cysteine allowed us to apply the substituted cysteine accessibility method (SCAM) [156] to probe their solvent accessibility. We observed that many of the residues in EL1 were not accessible to solvent despite their expected placement in the extracellular space according to the widely used and accepted snake diagram employed to represent GPCRs (see Figure 2). The accessibility of certain residues was altered by binding of agonist but not antagonist. These observations led to the hypothesis that certain Ste2p residues of EL1 are not accessible to solvent due to their interaction with residues in this GPCR’s N-terminal domain. Such interactions may play a role in the transition of Ste2p from an inactive to active state.

We extended the SCAM analysis to the Ste2p N-terminus [153], again finding that only certain amino acid residues in the N-terminus were accessible to solvent. Moreover, some of the Cys-substituted mutants formed dimers indicating that the N-terminus could be considered to be a dimer-inducing domain. These data supported the conclusions of previous experiments that the N-terminus of Ste2p is structured. Thus, in the absence of X-ray, cryoelectron microscopy, or nuclear magnetic resonance analysis, biochemical data provided insights into structural aspects of Ste2p function.

#### 7.2.2. Transmembrane Domains

Most studies on GPCRs have found that upon ligand binding the transmembrane bundle transduces the conformational transition of a GPCR from an inactive to an active state. Although above we suggested that the N-terminus appears to be involved in this transition, we are aware that residues in the transmembrane domains are central to signal propagation by Ste2p. This was elegantly demonstrated by a number of studies predominantly from the Konopka laboratory [40,127,129,157].

Noting that Y266 at the extracellular end of TM6 was involved in signal transduction, we initiated biological and biochemical analyses of wild-type and site-directed mutant receptors to search for interacting residues. We identified Asn205 as a potential interacting partner with the Tyr266 [158]. An N205H/Y266H double mutant showed pH-dependent functional activity, whereas the N205H receptor was non-functional and the Y266H receptor was partially active indicating that the histidine 205 and 266 residues interact in an activated state of the receptor. The introduction of N205K or Y266D mutations into the P258L/S259L constitutively active receptor suppressed the constitutive activity; in contrast, the N205K/Y266D/P258L/S259L quadruple mutant was fully constitutively active, indicating again an interaction between residues at the 205 and 266 positions in the receptor-active state. Using cysteine crosslinking and trypsin digestion, evidence was found that N205C and Y266C interacted in the constitutively active but not inactive state [158]. This study represented the first experimental demonstration of an interaction between specific residues in an active state, but not the resting state, of Ste2p. These studies led us to explore the interactions among other residues in TMs in the inactive and active states of Ste2p

Disulfide cross-linking experiments revealed that residues near the cytoplasmic ends of helices 5 (TM5) and 6 (TM6), which flank the amino and carboxyl sides of IL3, undergo conformational changes upon ligand binding. In contrast, those in the center of the IL3 loop do not [159]. Alpha-factor-induced Ste2p activation reduced cross-linking mediated at the cytoplasmic ends of TM5 and TM6 but not by residues in the middle of IL3. However, an α-factor antagonist desTrp^1^, desHis^2^-α-factor did not influence disulfide-mediated Ste2p cross-linking. These findings suggested that the interaction of the N-terminus of α-factor with Ste2p is critical for inducing conformational changes at TM5 and TM6. This study also showed that single Cys substitution of residues in the middle of IL3 led to receptors that appeared to form high levels of Ste2p dimers, as judged by increases in the band intensity at the dimer position on polyacrylamide gels. The high molecular weight band was not a Ste2p-Gα heterodimer. However, these data did not rule out the possibility that the high molecular band was actually a complex of Ste2p with an unidentified cellular protein. An alternating pattern of residues involved in cross-linking suggested the presence of a 3_10_-helix in the middle of IL3.

The importance of dimerization for the function of GPCRs was once the subject of much debate. Ste2p as a model GPCR has provided information relative to this issue. The dimerization involving the N-terminus and IL3, noted in the previous paragraphs, was unusual for GPCRs. Most studies have implicated the role of TMs [160,161] in this phenomenon. Transmembrane domains one (TM1) and four (TM4) of Ste2p were shown previously to play a role in dimerization of Ste2p [162]. We introduced single cysteine substitutions in residues in TM1 (L64 to M69) and TM7 (T278 to A296) to create 25 single Cys-containing Ste2p molecules [163]. Ste2p mutant V68C in TM1 and five mutants in TM7 (C278, C285, C289, and C291 to C296) showed increased dimerization upon addition of an oxidizing agent in comparison to the background dimers formed by the Cys-less receptor. The formation of dimers was decreased for TM7 mutant receptors in the presence of α-factor indicating that ligand binding resulted in a conformational change that influenced dimerization. The effect of ligand on dimer formation suggested that dimers formed in the resting state and the activated state of Ste2p involve different TM interactions. In addition, BRET (Bioluminescence Resonance Energy Transfer) analysis was used to study Ste2p oligomerization and showed that full-length Ste2p dimerized and that signaling required interaction between two functional monomers [164]. Receptor dimerization involving a truncated, defective receptor and a full-length, functional receptor leads to loss of signaling; this phenomenon is referred to as a dominant-negative effect as shown in mammalian GPCRs [165]. In an as yet to be published study, we have demonstrated that bound α-factor labeled separately with green and red fluorophores exhibited FRET interactions. In principal these experiments can be used to determine the distance between ligands in the monomer binding site and perhaps distinguish between bound agonists and antagonists (M. Dumont and F. Naider, unpublished results).

The G-protein signaling pathway involves the induction of a conformational change in Gα by the activated receptor. We used BRET to investigate the interaction between Ste2p and Gpa1p, the yeast Gα protein. The N-terminal residues of (Gpa1p) were conjugated to a donor bioluminescent reporter and the C-terminal residue of a full-length, or C-terminally truncated Ste2p, was conjugated to an acceptor bioluminescent reporter [166]. This methodology permitted the interrogation of the interaction between Gpa1 and Ste2p and allowed us to conclude that Ste2p and Gpa1p pre-couple before activation, as has been observed for many GPCRs [167]. Using confocal fluorescence microscopy and a bimolecular fluorescence complementation (BiFC) assay, we showed that a Ste2p-Gpa1p heterodimer assembled during biosynthesis on the endoplasmic reticulum and on the plasma membrane. These data were the first to demonstrate in vivo the formation of a super-structural complex between Gpa1p and Ste2p prior to membrane assembly and receptor activation.

#### 7.2.3. The C-Terminal Domain

The role(s) of the C-terminus of Ste2p were explored by the Konopka lab, showing that truncation of the C-terminal 105 amino acids resulted in defective endocytosis [168] and the promotion of the formation of receptor-G-protein pre-activation complexes without affecting ligand-induced signaling [169]. The signaling and internalization of Ste2p were reported to be regulated by the phosphorylation status of serine (S) and threonine (T) residues located in the cytoplasmic C-terminus [105,170]. We undertook a systemic analysis of all the S/T residues that are spread throughout the C-terminus [107]. Point mutations to alanine were introduced into the S/T residues located in the C-terminus individually or in combination. A series of functional assays such as Ste2p internalization, α-factor-induced gene activation, and growth arrest were conducted comparing WT and Ala-substituted Ste2p. Yeast harboring Ste2p in which all S/T residues in the C-terminus were mutated to alanine was more sensitive to α-factor, suggesting that phosphorylation in the C-terminus exerts negative regulation on the Ste2p signaling. Serine and threonine residues in the C-terminal tail that were within 10 residues of the C-terminus of the seventh transmembrane domain were important for ligand-induced G protein coupling, but not for receptor internalization. Sites in the central region of the C-terminus regulated both constitutive and ligand-induced internalization. Residues on the distal part of the CT (C-terminal tail) were important for constitutive desensitization and modulated G protein-signaling. This study demonstrated that the C-terminus contained multiple functional domains in which the S/T residues play differential and interdependent roles in regulating Ste2p function

GPCRs are all characterized by a 7-helix bundle, yet an additional helix named the 8th helix, located in the C-terminus proximal to the seventh transmembrane domain, has been recognized [102,171,172,173]. This eighth helix domain (H8) contains signature amino acids that have been implicated in multiple receptor functions from ligand binding to endocytosis. As mentioned above our biophysical analyses of TM7-CT peptides identified this eighth helix in the presence of micelles. In unpublished work we found that H8 of Ste2p plays a crucial role in the signaling activity of the receptor and in its ability to bind α-factor. We performed alanine-scanning mutagenesis of the H8 and identified residues that are important for receptor trafficking to the cell surface (T313, S315, T316), α-factor binding (D317, R318, S325) and signal transduction (S310, D311, T314, Y320, P321, T323, S326). We confirmed that Ste2p’s helix 8 has a DRY motif (residues D317, R318, Y320). For many class A GPCRs, there is a highly conserved DRY motif located at the boundary of TM3 and IL2 (intracellular loop 2, see Figure 2) that is involved in G protein interaction [174]. That the helix 8 motif in GPCRs interacts as well with G protein, despite an absence of the DRY motif, was the subject of a study by Sato et al. [173]. It is interesting that a DRY motif of Ste2p is found in helix 8. Thus, H8 plays multiple roles in Ste2p activity, and the study of its function may shed light on the structure and function of this structural element of medically important mammalian GPCRs.

## 8. Summary and Conclusions

Our goal in writing this review was to consider the use of biochemical, biophysical, and genetic methods in understanding the structural details of a model GPCR (Ste2p) interacting with and being activated by cognate peptide ligands. This paradigm has been our passion for the last 40 years. As we write, there is still no high-resolution model of Ste2p available, and all structural insights are based on the indirect methods surveyed in this chapter. The “high resolution” models of Ste2p without its bound ligand [175] and Ste2p bound to α-factor are based on homology modeling, using rhodopsin as a template and biochemical/mutagenesis data as constraints for α-factor binding [176]. Rhodopsin and Ste2p have very little homology and differ significantly in the extracellular N-terminus and the length of the extracellular loops [175]. Activation by light or by binding of a 13-residue peptide are expected to require quite distinct interactions with the receptor.

**Figure 9:**
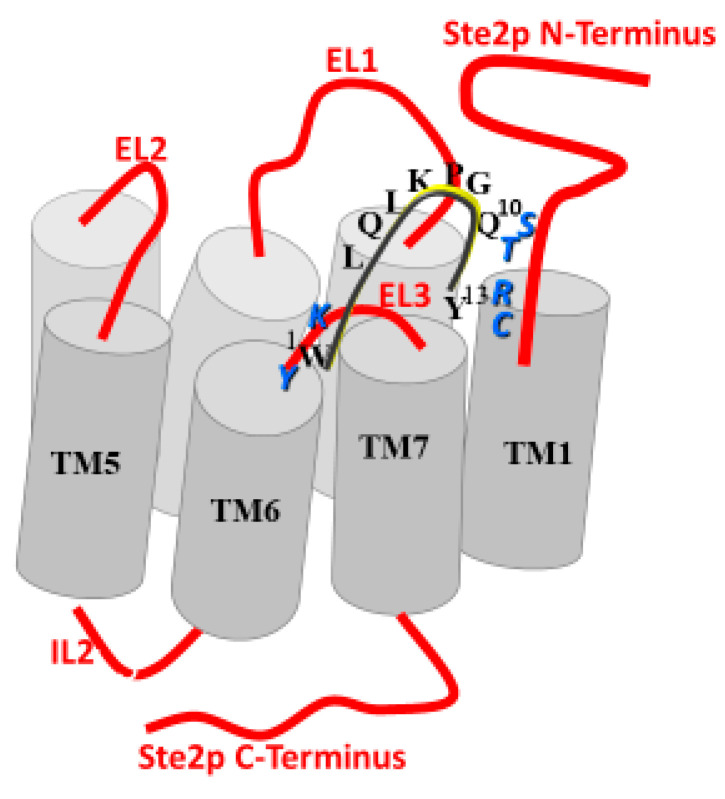
Binding of α-factor to Ste2p. Glutamine (Q^10^) of the C-terminus of α-factor binds to residues Ser47 and Thr48 at the interface of the receptor’s N-terminus and the extracellular face of TM1 and tyrosine (Y^13^) binds to Arg58 and/or Cys59 in TM1. The bending of the pheromone around the loop region facilitates the interaction between the tryptophan (W^1^) at the N-terminus of α-factor and residues Y266 and K269 in TM6 and EL3. The N-terminus is shown interacting with EL1. α-Factor residues are indicated in black (not all the residues of the pheromone are shown in the figure), Ste2p residues are shown in blue, Ste2p loops and termini are in red, and transmembrane domains are in gray.

The model of α-factor bound to Ste2p of Robles et al. [176] and our conclusions concerning the structure of bound α-factor (Figure 9) based on experimental results, without reliance on the rhodopsin model, have a high degree of similarity, as might be expected due to the fact that Robles et al. used contacts between α-factor and Ste2p derived from our experimental data.

In the Robles et al. model, the N-terminus of α-factor is found to interact with residues such as Y266 and K269, which are at the boundary between TM6 and EL3. This is consistent with mutagenesis and crosslinking data. The model has interactions of residues 47 and 48 with the Gln^10^ residue of α-factor consistent with site-directed mutagenesis. In addition, among the 26 residues in their modeled binding pocket are Ser108 and Thr114, identified in our SCAM analysis as critical for ligand interaction and signal transduction. The only significant conflict between our data and the Robles model is the interaction between Tyr^13^ of α-factor and Ste2p. Whereas we have two independent crosslinking studies that indicate Tyr^13^ interacts with residues in TM1 (Arg58 and Cys59) and a third that shows BPA in place of F55 can crosslink to α-factor, in the description of the modeling and the docking of α-factor into their Ste2p model, Robles et al. input a Tyr^13^-F204 interaction. Therefore, it is not surprising that they do not find the interaction of Tyr^13^ with TM1. These small discrepancies may be due to the ground-state rhodopsin template they used as the model for the receptor; crosslinking studies with agonists presumably capture the activated state of Ste2p. The resolution of this apparent contradiction will come from a high-resolution structure of Ste2p bound to agonists and antagonists. It would have been interesting to see what Robles et al. would have found if the Tyr^13^-F204 constraint had been replaced with a Tyr^13^-Arg58 constraint.

Our studies on the structure and function of α-factor analogs showed that their undefined structure in solution undergoes a conformational change as it binds to Ste2p. It was surprising that this relatively short tridecapeptide had regions predominantly involved in binding (the 3 C-terminal residues of the pheromone), a loop or bend region (that did not play a critical role in binding or signaling), and the four amino acid N-terminus that was required to initiate signal transduction.

We found that studies on fragments of Ste2p revealed important information on the secondary structure of the different sequences this GPCR, including transmembrane domains, the N-terminus, loop regions, and the C-terminus. The ability of NMR studies to provide information about transient helices such as the amphiphilic helices in the N-terminus of Ste2p and NY4 and the 8th helix in the C-terminus of Ste2p can help explain the role of these regions in ligand recognition, receptor regulation, and signal transduction. Such NMR studies can be applied to other GPCRs.

Our experiments led to a number of unexpected findings: the N-terminus had structural sub-domains, it interacted with specific sub-domains of the EL1 domain, and it played a role in Ste2p conformational change upon ligand binding. Very few GPCR X-ray structures include the N-terminus due to the inherent flexibility of this GPCR domain. Thus, biochemical and biophysical investigations of the N-terminus in the intact receptor and on receptor peptide surrogates, respectively, provide complementary information to crystallography [177,178].

Furthermore, our studies showed interactions between specific residues of Ste2p in an active state, but not resting state, and the effect of ligand on dimers formed in the inactive and active state of Ste2p. Crystals of GPCRs represent static states of a receptor, and may not capture different functional interactions during receptor activation. In conclusion, we strongly posit that by using a battery of different biochemical approaches, deep insight can be gained into the structure and conformational dynamics of GPCR-peptide interactions.
